# Renal Atrophy Following Selective Transcatheter Arterial Embolization for Angiomyolipoma as an Uncommon but Significant Complication: A Case Report and Literature Review

**DOI:** 10.7759/cureus.81968

**Published:** 2025-04-09

**Authors:** A B Azharul Islam, Samuel Bishara, Konstantinos Charitopoulos, David Ellis, Panagiotis Nikolinakos, Maisha Zaman Poushi, Ivo Donkov

**Affiliations:** 1 Urology, West Middlesex University Hospital, London, GBR; 2 Surgery, Dhaka Medical College and Hospital, Dhaka, BGD

**Keywords:** chronic kidney damage, glomerular filtration rate (gfr) decline, renal angiomyolipoma (aml), renal atrophy, renal infarction, transcatheter arterial embolization (tae)

## Abstract

Renal angiomyolipoma (AML) is a benign mesenchymal tumor that is often treated with transcatheter arterial embolization (TAE) to prevent complications such as hemorrhage. Although TAE is generally effective, it can lead to complications, including renal ischemia and atrophy. We present the case of a 72-year-old woman who underwent embolization for a right renal AML. After the procedure, she experienced damage to the renal artery, which resulted in progressive kidney shrinkage and impaired renal function. This case highlights the potential complications of embolization in managing renal AML and highlights the necessity for the long-term monitoring of renal function after the procedure.

## Introduction

Renal angiomyolipoma (AML) is a benign tumor of the kidney that comprises abnormal blood vessels, smooth muscle, and fat tissue. It accounts for 2-6% of all kidney tumors [1]. Pathologically, AML is more accurately classified as a perivascular epithelioid cell neoplasm [2]. Although most renal AMLs occur sporadically, some are associated with tuberous sclerosis complex (TSC). The overall incidence of sporadic AMLs is approximately 0.44%, with rates of 0.60% in females and 0.28% in males [3,4]. The abnormal blood vessels in AMLs are fragile and prone to rupture due to the replacement of smooth muscle with fibrous tissue and the lack of an internal elastic lamina [5].

Most of these tumors are often found incidentally during radiological imaging; however, symptomatic presentations like flank pain, gross hematuria, or severe retroperitoneal hemorrhage may also occur [6].

The primary diagnostic feature of classic AML is the presence of a significant amount of adipose tissue observed through radiological imaging. Although these tumors are typically benign, they can invade the surrounding perirenal fat, the renal sinus, and nearby organs and lymphatic structures [7,8].

Management of AML is guided by factors such as clinical symptoms, tumor size, quantity, growth pattern, and potential for malignancy. For example, epithelioid AML of the kidney, a rare subtype of AML, is considered potentially malignant [9].

We present the case of a 72-year-old woman who developed renal atrophy as a rare complication following transcatheter arterial embolization (TAE) for AML. This case highlights the importance of meticulous embolization techniques to prevent renal ischemia. We also review the literature to highlight the clinical implications of this uncommon complication.

## Case presentation

Background

A 72-year-old woman with hypertension and hyperparathyroidism was referred to our outpatient clinic after an incidentally detected right renal mass during an abdominal ultrasound for vague right flank pain. She had no history of tuberous sclerosis (TS), previous urological procedures, or family history of renal disease. Physical examination was unremarkable apart from elevated blood pressure (164/91 mmHg).

Initial findings

Imaging

**Figure 1:**
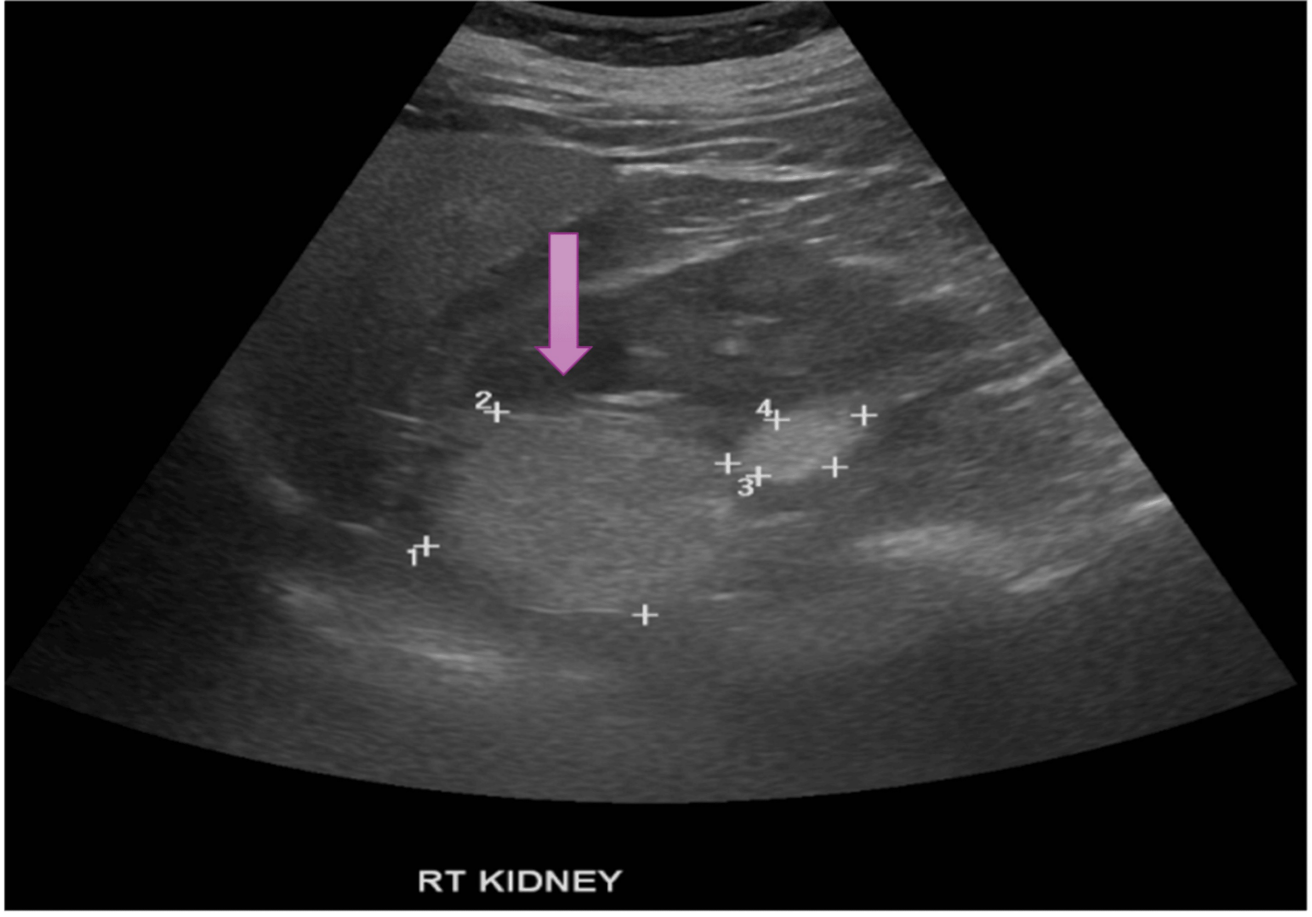
Pre-procedure USG KUB: The purple arrow indicates a hyperechoic cortical nodule in the right kidney. The numbered markers (1-4), placed by the sonographer, represent caliper measurements of the lesion's dimensions and were intended for size documentation. USG KUB: ultrasound of the kidneys, ureters, and bladder

Ultrasound showed a 117 mm right kidney with a hyperechoic cortical nodule (Figure 1).

**Figure 2:**
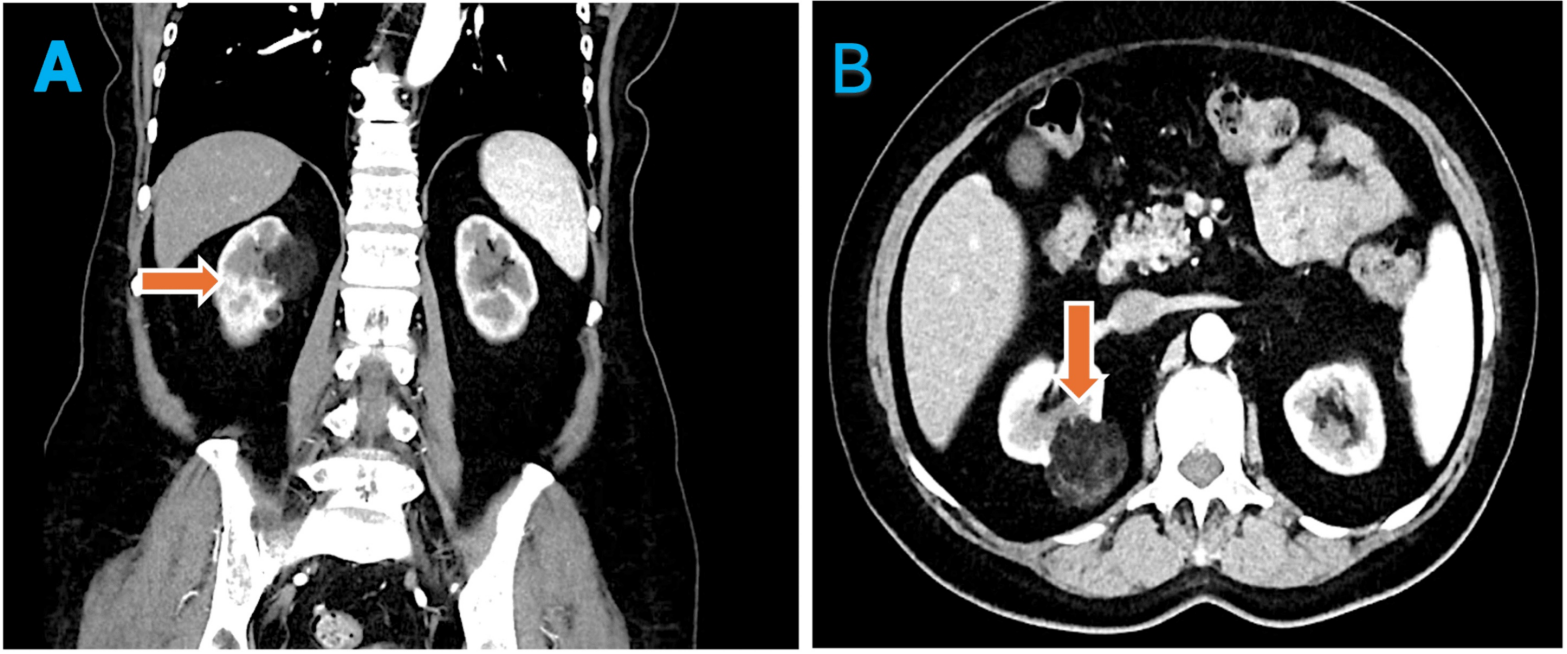
Pre-procedure CT angiogram. (A) Coronal section: Orange arrow indicates the normal-sized right kidney. (B) Axial section: Orange arrow highlights the right renal angiomyolipoma.

A CT angiogram revealed a 5.3 cm right renal AML with a feeding artery from an early branch of the mid-right renal artery. No active hemorrhage was detected (Figure 2).

Lab Results

Table 1 displays the patient's pre-procedure laboratory findings.

**Table 1:** Pre-procedure laboratory findings eGFR: estimated glomerular filtration rate; Hb: hemoglobin

Parameter	Value	Reference range
eGFR	61 mL/min/1.73 m²	>89 mL/min/1.73 m²
Creatinine	82 umol/L	55-110 umol/L
Hb	136 g/dL	114-150 g/dL
Serum calcium	2.31 mmol/L	2.20-2.60 mmol/L

Intervention and outcome

**Figure 3:**
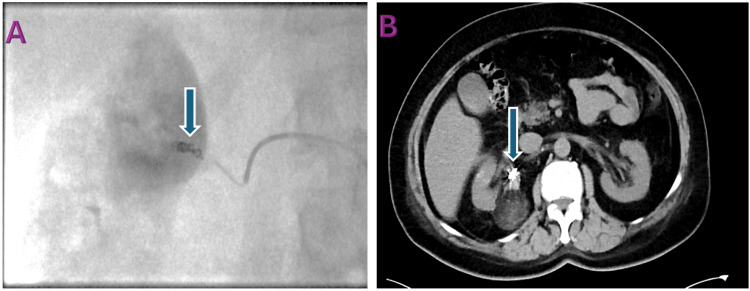
Intraoperative and post-procedure imaging. (A) Intraoperative fluoroscopic angiography: Blue arrow indicates the micro nester coil. (B) Postoperative CT angiogram: Blue arrow highlights the embolization coil.

Due to the AML size and bleeding risk, selective TAE was on the feeding vessel using 355-500 micron polyvinyl alcohol (PVA) particles and micro nester coil. The procedure was technically successful, resulting in the immediate devascularization of the tumor. Completion angiography confirmed the satisfactory exclusion of the right renal AML (Figure 3).

Follow-up

**Figure 4:**
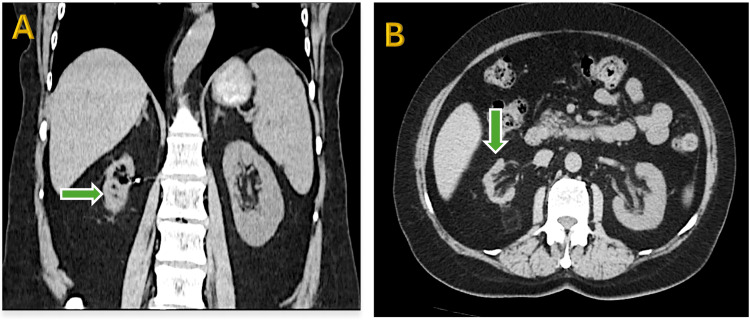
Follow-up CT of the kidneys showing right renal atrophy. (A) Coronal section: Green arrow indicates the atrophic right kidney. (B) Axial section: Green arrow highlights the atrophic right kidney.

At six months, renal function declined (estimated glomerular filtration rate (eGFR) from 61 to 48 mL/min/1.73m²) with CT evidence of post-embolization ischemic atrophy (Figure 4).

At the next three-month follow-up, despite conservative management with antihypertensive therapy, eGFR further dropped to 31 mL/min/1.73m², and a follow-up scan showed progressive kidney shrinkage.

## Discussion

Renal AML is a benign mesenchymal tumor characterized by varying proportions of dysmorphic blood vessels, fat, and smooth muscle components.

Selective arterial embolization (SAE) has emerged as an effective treatment for reducing tumor size and preventing bleeding in renal AML cases. Its minimally invasive nature and lower risk of serious complications compared to surgery have led to its increased use as a preventive therapy for AML [10,11]. Embolization of AML has been performed using various materials, including PVA particles, micro coils, gelatin sponge, ethanol, and ethiodized oil. These materials are commonly used to achieve complete embolization of the distal AML vascular bed [12,13].

Hemorrhaging associated with renal AMLs can pose a significant risk to patient health. Therefore, it is standard practice to initiate treatment for individuals who exhibit symptoms or have tumors that exceed 4 cm in size [14]. A retroperitoneal hematoma is the most serious complication of nontraumatic rupture of AML. The risk of rupture is primarily linked to intratumorally aneurysms and tumor size exceeding 4 cm. Other factors include coagulopathies, hormone levels, pregnancy, trauma, and associations with TS and lymphangioleiomyomatosis [15].

Due to the rare occurrence of renal atrophy following TAE for AML and the limited literature available on this subject, we conducted a review of related studies and case reports. A study by Kothary et al. found that while TAE effectively controls tumor growth and prevents hemorrhage, careful technique is essential to protect healthy renal tissue [12]. In a case reported in UroToday International Journal, a 33-year-old woman developed renal atrophy after embolization for renal hemorrhage [16]. Additionally, a 2020 study by Anis et al. noted a case where thrombosis of the main renal artery following selective embolization led to renal ischemia and complete loss of renal function [17]. Tomita et al. highlighted that using micro-balloon catheters during TAE can improve precision, thus decreasing the chance of harming healthy renal tissue and preventing subsequent atrophy [18].

Despite advances in embolization techniques, the risk of renal infarction from non-target embolization remains a significant concern. In this case, damage to the renal artery after embolization resulted in considerable renal shrinkage, highlighting the necessity for accurate embolization methods and careful patient selection. Monitoring renal function over the long term after embolization is essential for the early detection of any progressive deterioration in kidney health.

## Conclusions

This case highlights the risk of renal artery damage after embolization, which can cause kidney shrinkage in AML patients. While TAE is effective in controlling AML growth and preventing hemorrhage, careful technique is crucial to minimize damage to healthy renal tissue. Clinicians should minimize ischemic injury during treatment and regularly assess renal function to optimize patient outcomes.
